# ATM haplotypes and breast cancer risk in Jewish high-risk women

**DOI:** 10.1038/sj.bjc.6603062

**Published:** 2006-04-04

**Authors:** M Koren, G Kimmel, E Ben-Asher, I Gal, M Z Papa, J S Beckmann, D Lancet, R Shamir, E Friedman

**Affiliations:** 1 Susanne Levy Gertner Oncogenetics Unit, The Danek Gertner Institute of Human Genetics, Tel-Hashomer, Israel; 2 The Faculty of Exact Sciences, School of Computer Science, Tel-Aviv University, Tel-Aviv, Israel; 3 Department of Molecular Genetics and the Crown Center for Human Genome, Weitzman Institute of Science, Rehovot 76100, Israel; 4 Department of Oncological Surgery, The Chaim Sheba Medical Center, Tel-Hashomer, Israel; 5 Sackler School of Medicine, Tel-Aviv University, Tel-Aviv, Israel

**Keywords:** ATM gene, breast cancer risk, SNP, haplotypes, high-risk populations, Jewish breast cancer patients

## Abstract

While genetic factors clearly play a role in conferring breast cancer risk, the contribution of ATM gene mutations to breast cancer is still unsettled. To shed light on this issue, ATM haplotypes were constructed using eight SNPs spanning the ATM gene region (142 kb) in ethnically diverse non-Ashkenazi Jewish controls (*n*=118) and high-risk (*n*=142) women. Of the 28 haplotypes noted, four were encountered in frequencies of 5% or more and accounted for 85% of all haplotypes. Subsequently, ATM haplotyping of high-risk, non-Ashkenazi Jews was performed on 66 women with breast cancer and 76 asymptomatic. One SNP (rs228589) was significantly more prevalent among breast cancer cases compared with controls (*P*=4 × 10^−9^), and one discriminative ATM haplotype was significantly more prevalent among breast cancer cases (33.3%) compared with controls (3.8%), (*P*⩽10^−10^). There was no significant difference in the SNP and haplotype distribution between asymptomatic high-risk and symptomatic women as a function of disease status. We conclude that a specific ATM SNP and a specific haplotype are associated with increased breast cancer risk in high-risk non-Ashkenazi Jews.

Inherited predisposition to breast cancer is well established in *BRCA1* (MIM# 113705) and *BRCA2* (MIM# 600185) mutation carriers (reviewed by Narod and Foulkes, 2004). Yet, only 20–40% of familial inherited breast cancer risk is conferred by *BRCA1/2* mutations, and clearly other genes are involved in familial breast cancer clustering (Ford *et al*, 1998; Nathanson and Weber, 2001; Thompson and Easton, 2004; Garber and Offit, 2005). A strong candidate for a breast cancer predisposition gene is ATM (MIM# 607585). The attribution of ATM candidacy as a breast cancer susceptibility gene stems from two sources. Functionally, the ATM protein is a pivotal player in mediating cellular responses to DNA damage, including DNA double-strand break repair and signaling, leading to cell-cycle arrest and apoptosis (reviewed in Rotman and Shiloh, 1999). From the genetic perspective, ATM is the gene mutated in ataxia-telangiectasia (AT), an autosomal recessive disorder phenotypically characterised by chromosomal instability and an increased risk for lymphoproliferative tumors in homozygotes (Swift *et al*, 1991; Gatti *et al*, 1999). Ataxia-telangiectasia heterozygotes that are asymptomatic have been reported to be at an increased risk for developing breast cancer (Swift *et al*, 1991; Gatti *et al*, 1999; reviewed by Hall, 2005), although these reports are still controversial and not universally accepted. ATM gene's contribution to breast cancer risk was previously evaluated in the context of high-risk families, in *BRCA1/2* mutation carriers, and in average risk populations (reviewed in Gatti *et al*, 1999; Hall, 2005). The results of these studies are inconclusive, with some studies reporting an increased breast cancer risk (Swift *et al*, 1987; Pippard *et al*, 1988; Athma *et al*, 1996; Stancovic *et al*, 1998; Inskip *et al*, 1999; Janin *et al*, 1999) and others failing to demonstrate such an effect in heterozygote ATM mutation carriers (Vorechovsky *et al*, 1996; FitzGerald *et al*, 1997; Bay *et al*, 1998; Chen *et al*, 1998).

In order to shed further light on the putative contribution of ATM to breast cancer risk, we performed haplotyping of the ATM locus in high-risk individuals and controls of non-Ashkenazi Jewish origin.

## MATERIALS AND METHODS

### High-risk participants: identification, recruitment, and data collection

All high-risk individuals studied herein were ascertained and identified from among individuals referred for genetic counseling and testing at the Oncogenetics unit, Sheba Medical Center, Tel Hashomer Israel. Only one individual per high-risk family was included in the study. All participants were counseled for family history of breast cancer, and all affected women had histopathologically proven breast cancer. Relevant demographic and clinical data were collected at the time of initial genetic counseling and included type of malignancy (based on pathology reports), age at diagnosis, age at counseling, and ethnic origin at least three generations back. High risk was assigned based on current accepted criteria (Lynch and Lynch, 2002). The study was approved by the institutional review board (Helsinki committees) at Sheba Medical Center, and each participant signed a written informed consent. Based on the results of the genetic testing (see below), none of the study participants was a carrier of any of the predominant Jewish mutations in *BRCA1/2*.

### Control population

DNA samples were taken from unrelated, healthy, non-Ashkenazi individuals, with no personal or familial history of cancer. These were recruited primarily from among women who escorted the high-risk women but were unrelated to them (e.g. friends, married ins, etc.). All study participants among controls gave their consent for anonymous testing.

### Origin of patients

All the patients in this study are non-Ashkenazi Jews originating from Iraq, the Balkan, and Yemen.

### DNA isolation

Genomic DNA was prepared from anticoagulated, venous blood samples using the PUREGene DNA isolation kit (Gentra systems Inc., Minneapolis, MN, USA) using the manufacturer's recommended protocol.

### Genotyping for the recurring BRCA1/BRCA2 mutations

None of the (high risk and control) participants of this study carried any of the four recurring *BRCA1* (185delAG, 5382InsC, Tyr978X) and *BRCA2* (6174delT) mutations reported in Jewish individuals. Detection of these mutations was carried out by modified restriction enzyme digest assay, which distinguishes the mutant from the wild-type allele, using primer sequences, cycling profiles, PCR conditions, and gel electrophoresis as previously described (Rohlfs *et al*, 1997; Shiri-Sverdlov *et al*, 2001). Only individuals not carrying these mutations were included in the subsequent study.

### ATM SNP selection and genotyping

*ATM* genotyping was performed by PCR amplification of eight SNPs throughout the gene. The SNPs were chosen from three databases: www.ensembl.org www.genome.ucsc.edu and www.ncbi.nlm.nih.gov. The SNPs genotyped were ss4328153 (now rs3092993), rs228589, rs600931, rs664677, rs227069, rs664982, rs652541, and rs170548 (Table 1). SNP genotyping was carried out using the Sequenom™ MASSarray system (Sequenom, San-Diego, CA, USA).

### Sequenom™ MassARRAY system

PCR amplification was performed in 384-well microplates (Marsh Biomedical Products, Rochester, NY, USA), in a total volume of 5 *μ*l, using 2.5 ng reaction^−1^ DNA, 10 × PCR Buffer containing 1.5 mM MgCl_2_, 200 mM dNTPs mix, 0.02 *μ*l HotStar Taq Polymerase at 5U *μ*l^−1^ (Qiagen Inc., Valencia, CA, USA), and 1 pmol each of forward and reverse PCR primer. After an initial denaturation at 94°C for 5 min, 45 cycles of 94°C for 20 s, annealing at 56°C for 30 s, and extension at 72°C for 1 min were carried out with a final extension period of 3 min. Primer sequences, designed using the software SpectroDESIGNER (Sequenom, San-Diego, CA, USA), are shown in Table 2.

PCR amplification was performed in multiplex reactions as follows:

Multiplex 1: SNPrs228589+SNPrs3092993+SNPrs170548.

Multiplex 2: SNPrs652541+SNPrs227069+SNPrs664982.

Multiplex 3: SNPrs664677+SNPrs600931.

Following PCR, SNP genotyping proceeded as previously described (Little *et al*, 1997a, 1997b; Buetow *et al*, 2001).

Similar to these above-mentioned studies, quality control and quality assurance were provided by randomly including non-DNA containing well in the chip as well as re-genotyping about 10% of the samples for all SNPs on different chips.

### Statistical methods, haplotype reconstruction, and association analyses

The process of phasing the genotypes and imputing the missing data was performed by the software GERBIL (Kimmel and Shamir, 2005).

The association between sequence variants and breast cancer was evaluated by permutation test (Zhang *et al*, 2002) as follows: to evaluate the overall *P*-value of the association between the SNPs genotypes and the disease, the Pearson score of each marker is calculated, and the maximum value over all markers, denoted CC_max_, is chosen as the test statistic. Then, the same statistic is calculated for many data sets with the same genotypes and randomly permuted labels of the case and control individuals. The fraction of times that this value exceeds CC_max_ is used as the *P*-value. This test has the advantage of not assuming a specific distribution function. Additionally, it handles multiple-testing directly and avoids the bias of correction, for example, by the over-conservative Bonferroni method. This test was applied to cases *vs* controls, and also to the high-risk group *vs* controls.

Since there are three different population groups in the study, originating from Iraq, the Balkan and Yemen, the score for each marker was calculated as follows: Let *P*_*i*,*j*_ be the Pearson score of the *j*th marker for the *i*th population (out of three possibilities). The statistic CCP_max_ is defined to be max_*j*_∑_*i*_*P*_*i,j*_. The *P*-value is calculated by a permutation test as mentioned above, with the difference of randomly permuting the labels within each population independently, and using the statistic CCP_max_ instead of CC_max_. This statistic avoids the bias in the *P*-value that might occur due to the mixture of different populations.

The permutation test can be readily generalised to handle association between haplotypes and the disease, for example, by adding block haplotypes as artificial loci with states corresponding to common haplotypes. Similarly, one can add loci interactions as artificial loci, whose states are the allele combinations.

Estimates of odds ratio (OR), relative risk (RR), and 95% confidence interval (CI) were calculated for the discriminative SNPs and haplotypes (Tables 3A and 3B).

We repeated the above procedure to perform two different tests:

1. Association test for each SNP separately and for the haplotype composed from all the eight SNPs.
2. Association test of all possible pair-wise interactions of SNPs.

## RESULTS

### Characteristics of study participants

In total, 260 unrelated women of non-Ashkenazi Jewish origin were genotyped in this study. Of these, 142 were evaluated and considered at high risk for developing breast cancer based on their family history. Of the high-risk individuals, 66 (46.5%) were breast cancer cases (mean age at diagnosis was 48.3±9.7 years) and their ethnic origin was Iraqi (*n*=34 or 51.5%), Balkan (*n*=16 or 24.25%), or Yemenite origin (*n*=16 or 24.25%). Additional 76 (53.5%) women were asymptomatic, high-risk women. The age range at counseling for the high-risk asymptomatic group was 28–67 years (mean 50.3±10.5 years), and their ethnic distribution was as follows: 34 of Iraqi (44.7%), 26 of Balkan (34.2%), and 16 of Yemenite origin (21%). The ethnicity of the population-matched healthy controls (*n*=118) was 55 (46.61%) Iraqis, 29 (24.57%) of Balkan origin, and 34 (28.81%) Yemenites. The age range at counseling for the control group was 33–69 years (mean 53.4±8.9 years).

All participants were genetically prescreened and shown not carry any of the four common Jewish mutations in *BRCA1* (185delAG; 5382insC; Tyr978X) and in *BRCA2* (6174delT).

### Phasing the genotypes

Phasing the genotypes of the eight SNPs at the ATM locus yielded together 28 different haplotypes in one block of high LD (Table 4). Four haplotypes had a frequency ⩾0.05 (Table 5A) accounting together for 85% of all haplotypes. For association test of the haplotypes, we generated four clusters corresponding to the four common haplotypes and clustered each rare haplotype with the common haplotype to which it resembled most, as measured by Hamming distance.

### Association tests

We first compared the breast cancer patients to the control population. SNP 2 (rs228589) is the most associated with the disease, with score of 61.61 (after multiple testing correction: *P*=4 × 10^−9^). In this SNP, nucleotide T appears in 3.8% of the controls and in 33.3% of the cases (Table 3A). Odds ratios and RR values for this SNP are listed in Table 3B.

An association test was performed for each SNP separately and for the complete 8-SNP haplotype. The Pearson scores of association are presented in Table 6. Interestingly, this SNP alone shows higher association than the complete haplotype (score 31.45).

An additional test was performed for each pair of SNPs. The pair of SNPs most associated was SNP 1 (rs3092993) and 2 (rs228589) (both intronic SNPs) (*P*⩽10^−10^). We therefore examined the short genotypes consisting of SNP 1 and SNP 2. These genotypes form three common haplotypes, coined A, B and C (Table 5B), and additional rare haplotype of frequency 0.002, which we ignored for the association test. Haplotypes B and C were significantly more prevalent in cases (33.3%) compared with controls (3.8%): odd ratios and RR values for these two haplotypes are listed in Table 7. In agreement with the above, these two haplotypes were also significantly more prevalent in the healthy high risk (24.3 and 14.5%) compared with controls (3.8% and 4.7%). In contrast, haplotype A was significantly more prevalent in controls (91.5%) as compared to cases (56%) or healthy high risk (61.2%). Odd ratios and RRs followed compatible trends (Table 7A).

Next, we tested all case and high-risk patients clustered together as a single group *vs* controls. When testing each SNP separately, the most associated SNP is again SNP 2 (rs228589) (*P*=7 × 10^−9^). When testing all pairwise interactions of SNPs, the most associated pair is SNP 2 (rs228589) and SNP 3 (rs600931) (*P*⩽10^−9^) (Table 5C). One rare haplotype of frequency 0.006 was ignored for this test. One short haplotype consisting of SNPs 2 and 3, coined E (Table 7B) confers a RR of 7.2 95% CI (3.69–14.05), and an OR of 9.55 95% CI (4.67–19.5).

Testing association of the individual SNPs and of all the SNP pairs on the group of high-risk women *vs* cases yields no significant result (*P*=0.35).

## DISCUSSION

In this study, several ATM SNPs were seemingly associated with breast cancer risk in Jewish non-Ashkenazi women at high-risk for breast cancer. These results further establish ATM as a contributor to breast cancer susceptibility in high-risk populations.

Increased breast cancer risk in ATM heterozygote mutation carriers has been previously reported in studies that either inferred obligate carriership (Swift *et al*, 1987; Pippard *et al*, 1988) or directly tested for gene mutations (Athma *et al*, 1996; Stancovic *et al*, 1998; Inskip *et al*, 1999; Janin *et al*, 1999; Olsen *et al*, 2001). Epidemiological studies have consistently shown that female relatives of A-T patients are at an increased risk for developing breast cancer (reviewed in Hall, 2005). Interestingly, this increased risk was predominantly observed in the mothers of A-T carriers and not in siblings and offsprings (Olsen *et al*, 2005). Yet, not all studies confirmed the associated breast cancer risk conferred by being an ATM heterozygous mutation carrier (Vorechovsky *et al*, 1996; FitzGerald *et al*, 1997; Bay *et al*, 1998; Chen *et al*, 1998).

Two ATM germline alterations (Ala2524Pro and 6903insA) reported in A-T families have been shown to segregate with breast cancer in these families (Laake *et al*, 2000). Stancovic *et al* (1998) described two additional A-T families, where a heterozygous missense mutation, Val2424Gly (7271T<G) was associated with a presumed increased breast cancer risk. Another ATM mutation (IVS10-6T<G) was suggested to be associated with early-onset breast cancer risk in patients, who were exposed to low-dose ionising radiation (Broeks *et al*, 2000; Dörk *et al*, 2001). The latter two mutations were functionally shown to exert a dominant negative effect on ATM protein (Chenevix-Trench *et al*, 2002). The contribution of the Val2424Gly and the IVS10-6T<G mutations to increased breast cancer risk was further established in a large population-based, case–control study (Chenevix-Trench *et al*, 2002).

As most studies focused on sporadic rather than familial breast cancer cases, and employed screening methods preferentially capable of detecting protein-truncating mutations (Ángele and Hall, 2000), there might be more ATM non-truncating mutations and/or polymorphisms or variants (e.g. missense mutations) that affect breast cancer risk. In support of this notion, ATM missense substitutions seem to be more prevalent among Swedish, Canadian and Slovenian breast cancer patients (Dörk *et al*, 2001) and among US heterogeneous women (Teraoka *et al*, 2001). These findings give credence to the hypothesis that there are two distinct populations of ATM heterozygous mutations: null mutations or truncating mutations are not associated with breast cancer risk, whereas the presence of even a single missense allele may have a dominant negative effect on protein function and thus be associated with breast cancer risk (Meyn, 1999; Khanna, 2000). However, a more recent study from the UK (Thompson *et al*, 2005) shows that while being an ATM heterozygote does contribute to a modest increase in breast cancer risk, there are no differences in the risk as a function of mutation type.

The majority of studies conducted to assess ATM's contribution to breast cancer have used a variety of mutation detection techniques, with predominant bias for the detection of protein truncating mutations, or else they have examined the effect of specific ATM variants that are prevalent in the studied population (Hall, 2005). Only a handful of studies have used haplotyping, a mutation independent method, to assess the effect of ATM on breast cancer risk. Angele *et al* (2003) report that of the three major ATM haplotypes, one was significantly associated with breast cancer risk in French women. Similar results were also reported from Korea (Lee *et al*, 2005). Conversely, Tamimi *et al* (2004) used a large collection of cases and controls (more than 1300 individuals in each group) from the Nurses Health study, and report that none of five common ATM haplotypes was associated with breast cancer risk in American women.

The current study is the first to report ATM SNP and haplotype in a population of high-risk non-Ashkenazi Jewish women. Unlike the lack of a discriminating ATM haplotype among average risk Ashkenazi Jewish breast cancer women (Bonnen *et al*, 2001), the present study shows that ATM does contribute to familial clustering of breast cancer in non-Ashkenazim. It is noteworthy that specific genotypes are associated with breast cancer risk even without performing the phasing process. A very strong association (*P*=4 × 10^−9^) was noted by testing each SNP separately, and correcting for multiple hypotheses using permutation tests. Given the intronic position of the two SNPs most tightly associated with breast cancer risk and phenotype, it is unlikely that these SNPs in and by themselves are disease associated. Rather, in all likelihood they are in linkage disequilibrium with a pathogenic ATM mutation.

It is important to emphasise that only one patient was analyzed per high-risk family, so that patients in the high-risk group are not more genetically related to each other than in the control group. Additionally, our statistical method for computing the *P*-value takes into account the three different subpopulations and corrects for multiple testing. Hence, the strong association noted between the ATM genotype and the high-risk phenotype seems real, and cannot be accounted for as an artifact caused by analysis of related individuals.

The limitations of the study should be pointed out. This was a relatively small study that analyzed a highly selected population, and includes only non-Ashkenazi Jewish women who were recruited through high-risk clinic in a single medical center in Israel. Thus, the applicability of the results to average-risk population or even high risk, ethnically diverse populations, needs to be established.

In conclusion, the present study suggests that a specific ATM SNP seemingly contributes to breast cancer predisposition in Jewish non-Ashkenazi high-risk women in Israel.

## Figures and Tables

**Table 1:** ATM SNPs^a^

**SNP no.**	**SNP ID**	**Position^b^**	**Polymorphism**	**Minor allele frequency**
1	rs3092993^c^	11797531	A/C	0.068
2	rs228589	11655624	A/T	0.144
3	rs600931	11679751	A/G	0.236
4	rs664677	11705598	C/T	0.242
5	rs227069	11772674	A/G	0.272
6	rs664982	11787899	A/G	0.24
7	rs652541	11788441	C/T	0.155
8	rs170548	11797252	G/T	0.342

a All SNPs are intronic and noncoding.

b Position based on Genbank Accession Number NT_033899 (http://www.ncbi.nih.gov/entrez/query.fcgi?db=snp&cmd=search&term=rs).

c This SNP was originally coined ss4328153.

**Table 2:** Primer sequences used for detecting the relevant SNPs

**SNP**	**Forward, reverse primers**	**Extension primer**
1	F: ACGTTGGATGGTTAGCTGTTCTGAACTGCC	E: GAACTGCCAATATCAGAAATTC
	R: ACGTTGGATGGAGCAAGTAGCTTTAGGTCG	
2	F: ACGTTGGATGTTTGGCCTCAAAGGTCCTTC	E: GGGTCCAATAACCCTCC
	R: ACGTTGGATGCTTGTATTGGGTAAGCGCGG	
3	F: ACGTTGGATGCTCCGTATGCCTTTTTCTGG	E: TCTGGCCTAAGAGAAAAATATTAC
	R: ACGTTGGATGCTGAAATGGTGAGAAGTCTG	
4	F: ACGTTGGATGAGCACTCAGAAAACTCACTG	E: AAAACTCACTGAAAGGTTATT
	R: ACGTTGGATGGAGTATGTTGGCATATTCCAC	
5	F: ACGTTGGATGGCTGTGTACTTTCAGAGAAC	E: TCAGTCCTTTTTTGTGG
	R: ACGTTGGATGCTGGGTATCTGGGTATTTTG	
6	F: ACGTTGGATGCAGCATACTACACATGAGAG	E: CATGAGAGTATACAGATAAAGATA
	R: ACGTTGGATGCAGCATCTAGAGTCAAACAC	
7	F: ACGTTGGATGAGGTAGCACCAGCAGTAAAC	E: CCCTCATTCCTAAGCCA
	R: ACGTTGGATGGGAGATCAAATTGTCAGCATC	
8	F: ACGTTGGATGTTAATGGTCCTGGAGGACAC	E: CAAAACAGCATTAAAAAATAGAG
	R: ACGTTGGATGAGGACACGTACTAGATTAGC	

**Table 3A:** Frequencies of the most associated SNP (number 2, rs228589)

**Allele**	**Frequency**	**Case (*n*=66) %**	**Healthy high risk (*n*=76) %**	**Control (*n*=118) %**
A	0.856	66.7	51.4	96.2
T	0.144	33.3	48.6	3.8

**Table 3B:** Comparison of the most associated SNP (number 2, rs228589) between the study's subgroups: case *vs* control, and asymptomatic high-risk *vs* control

	**Alleles (%)**		**RR (95% CI)**		**OR (95% CI)**	
**Study subset**	**A**	**T**	**A**	**T**	**A**	**T**
Breast cancer case *vs* control	88 (67)	44 (33)	0.34 (0.27–0.42)	2.97 (2.4–3.69)	0.08 (0.04–0.17)	12.61 (5.91–26.92)
	227 (96)	9 (4)				

Asymptomatic high risk *vs* control	76 (51)	72 (49)	0.28 (0.23–0.35)	3.45 (2.87–4.37)	0.04 (0.02–0.09)	23.89 (11.4–50.08)
	227 (96)	9 (4)				

All high-risk women *vs* control	303 (79)	81 (21)	0.45 (0.4–0.51)	2.21 (1.95–2.51)	0.06 (0.03–0.11)	17.84 (8.79–36.19)
	227 (96)	9 (4)				

RR, relative risk; OR, odds ratio; CI, confidence interval.

**Table 4:** The LD scores (measured in *r*^2^) between all 8 SNPs

	**1**	**2**	**3**	**4**	**5**	**6**	**7**	**8**
1	−1	0.01	0.02	0.02	0.17	0.02	0.01	0.12
2	0.01	−1	0.49	0.50	0.41	0.51	0.88	0.17
3	0.02	0.49	−1	0.88	0.32	0.93	0.54	0.18
4	0.02	0.50	0.88	−1	0.29	0.87	0.57	0.19
5	0.17	0.41	0.32	0.29	−1	0.33	0.47	0.46
6	0.02	0.51	0.93	0.87	0.33	−1	0.58	0.19
7	0.01	0.88	0.54	0.57	0.47	0.58	−1	0.20
8	0.12	0.18	0.18	0.19	0.46	0.19	0.20	−1

**Table 5A:** The inferred haplotypes and their frequency

**Haplotype**	**Frequency**	**Haplotype sequence**							
**1**	**0.553**	**C**	**A**	**A**	**T**	**G**	**A**	**C**	**G**
**2**	**0.130**	**C**	**T**	**G**	**C**	**A**	**G**	**T**	**T**
**3**	**0.086**	**A**	**A**	**A**	**T**	**A**	**A**	**C**	**T**
**4**	**0.075**	**C**	**A**	**A**	**T**	**G**	**A**	**C**	**T**
5	0.032	C	T	G	C	A	G	T	G
6	0.025	C	A	G	C	G	G	C	G
7	0.017	C	A	A	T	A	A	C	T
8	0.017	C	A	G	C	G	G	C	T
9	0.008	C	A	G	C	A	G	C	T
10	0.008	C	A	A	T	A	A	C	G
11	0.008	C	A	G	C	A	G	T	T
12	0.004	C	A	G	T	A	G	C	T
13	0.004	C	A	A	T	G	G	C	G
14	0.004	C	A	G	C	G	A	C	G
15	0.002	C	A	A	C	A	A	C	T
16	0.002	C	T	G	T	A	G	C	T
17	0.002	C	A	A	C	G	A	C	G
18	0.002	C	A	G	C	A	G	C	G
19	0.002	A	A	A	T	G	A	C	G
20	0.002	A	T	A	C	A	G	T	T
21	0.002	C	T	G	C	A	G	C	G
22	0.002	C	A	A	T	G	G	C	T
23	0.002	C	A	A	C	G	A	C	T
24	0.002	C	A	G	T	A	G	C	G
25	0.002	A	A	A	T	A	A	C	G
26	0.002	C	A	A	C	A	G	T	T
27	0.002	C	T	A	T	G	A	C	T
28	0.002	C	T	A	C	G	G	T	T

Haplotypes that have frequency ⩾5% are indicated in bold.

**Table 5B:** Frequencies of the haplotypes composed of SNPs 1 and 2

**Haplotype**	**Haplotype sequence**	**Frequency**	**Case (*n*=66) %**	**Healthy high risk (*n*=76) %**	**Control (*n*=118) %**
A	CA	0.737	56	61.2	91.5
B	CT	0.171	33.3	24.3	3.8
C	AA	0.09	10.6	14.5	4.7

**Table 5C:** Frequencies of the haplotypes composed of SNPs 2 and 3

**Haplotype**	**Haplotype sequence**	**Frequency**	**Healthy high risk+case (*n*=142) %**	**Control (*n*=118) %**
D	AA	0.763	72.5	80.9
E	TG	0.167	27.5	3.8
F	AG	0.069	0	15.3

**Table 6:** Pearson scores for association of the individual SNPs and of the haplotype to the disease phenotype

**SNP**	**Score**
1	8.13
**2**	**61.61**
3	12.23
4	11.39
5	26.14
6	12.16
7	51.97
8	11.88
Haplotype	31.46

**Table 7:** Comparison of the short haplotypes between the study's subgroups: (A) case *vs* control, and asymptomatic high-risk *vs* control (Table 5B) and (B) all high-risk *vs* control (Table 5C)

	**Haplotypes (%)**			**RR (95% CI)**			**OR (95% CI)**		
**Study subset**	**A**	**B**	**C**	**A**	**B**	**C**	**A**	**B**	**C**
(**A**)									
Breast cancer case	74 (56)	44 (33)	14 (0.6)	0.61 (0.49–0.76)	8.74(3.32–23.02)	2.28 (0.78–6.67)	0.12(0.05–0.27)	12.61(4.32–36.84)	2.43(0.76–7.74)
*Vs* control	216 (91)	9 (4)	11 (4.6)						
Asymptomatic high risk	93 (61)	37 (24)	22 (14.5)	0.66 (0.55–0.81)	6.38 (2.37–17.16)	3.11(1.16–8.29)	0.146 (0.07–0.32)	8.115 (2.76–23.85)	3.461 (1.19–10.07)
*Vs* control	216 (91)	9 (4)	11 (4.6)						


(**B**)									
	**Haplotypes (%)**			**RR (95% CI)**			**OR (95% CI)**		
**Study subset**	**D**	**E**	**F**	**D**	**E**	**F**	**D**	**E**	**F**
All high-risk women	206 (73)	78 (27)	0 (0)	0.9 (0.82–0.99)	7.2 (3.69–14.05)	—	0.62 (0.41–0.94)	9.55 (4.67–19.5)	—
*Vs* control	191 (81)	9 (4)	36 (15)						

RR, relative risk; OR, odds ratio; CI, confidence interval.
